# Supplementary material for: JunB is essential for IL-23-dependent pathogenicity of Th17 cells
Source: Nat Commun. 2017 May 30;8:15628. doi: 10.1038/ncomms15628 (PMC5460000; doi:10.1038/ncomms15628)
Supplement: Supplementary Information — Supplementary Figures and Supplementary Table 1 [file ncomms15628-s1.pdf]

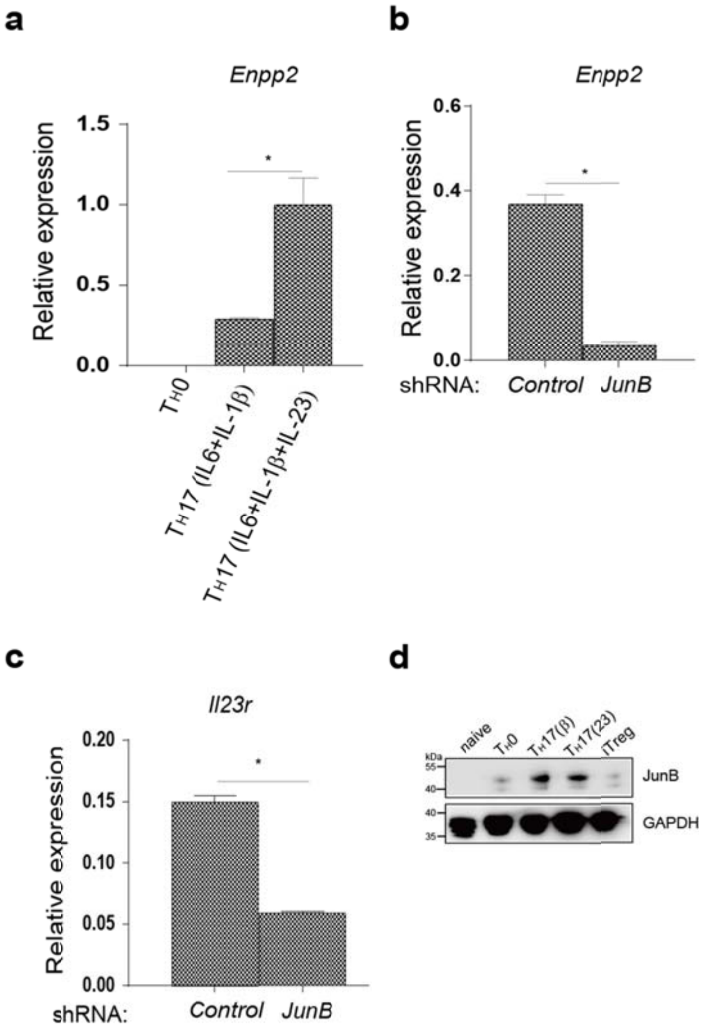

Supplementary Figure 1. (a) Naive CD4$^+$ T cells were activated in the presence of the indicated cytokines for 3 days. *Enpp2* mRNA expression was measured by qRT-PCR. (**b, c**) Naive CD4$^+$ T cells were activated under T_H0 condition for 36 hr, infected with retrovirus expressing JunB or control shRNA, and then incubated for another 3 days in the presence of IL-6, IL-1β, and IL-23. *Enpp2* (**b**) and *Il23r* (**c**) mRNA expression was measured by qRT-PCR. (**a-c**) Results were normalized to *Hprt* mRNA expression. Error bars indicate s.d. (n=3). Asterisks indicate significant differences ($p<0.05$) by unpaired two-tailed Student's t test. (**d**) Immunoblot analysis of JunB in naive or activated CD4$^+$ T cells under T_H0, $T_H17(\beta)$ and $T_H17(23)$ conditions for 60 hr. (**a-d**) Data represent two independent experiments.

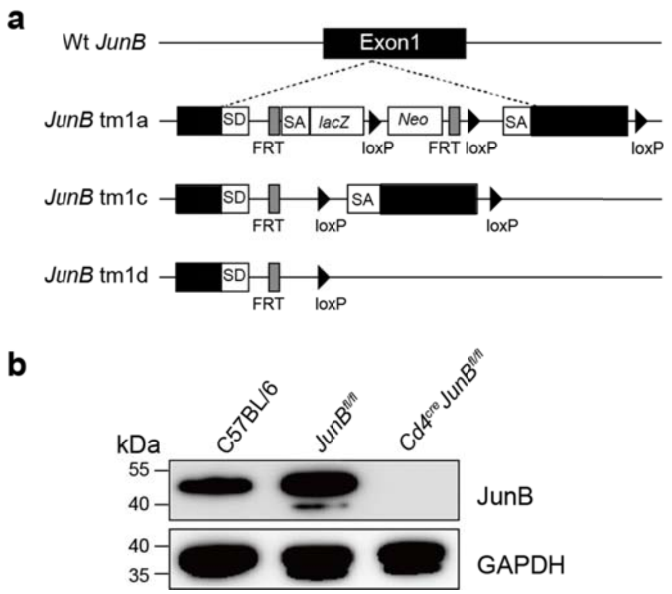

Supplementary Figure 2. (a) Schematic illustration showing the strategy to generate floxed *JunB* mice. (**b**) Naive CD4$^+$ T cells from wild-type C57BL/6, *Cd4^{Cre}JunB$^{fl/fl}$* and control *JunB$^{fl/fl}$* mice were activated under $T_H17(\beta)$ conditions for 3 days, and JunB was detected by immunoblot analysis. Data represent two independent experiments.

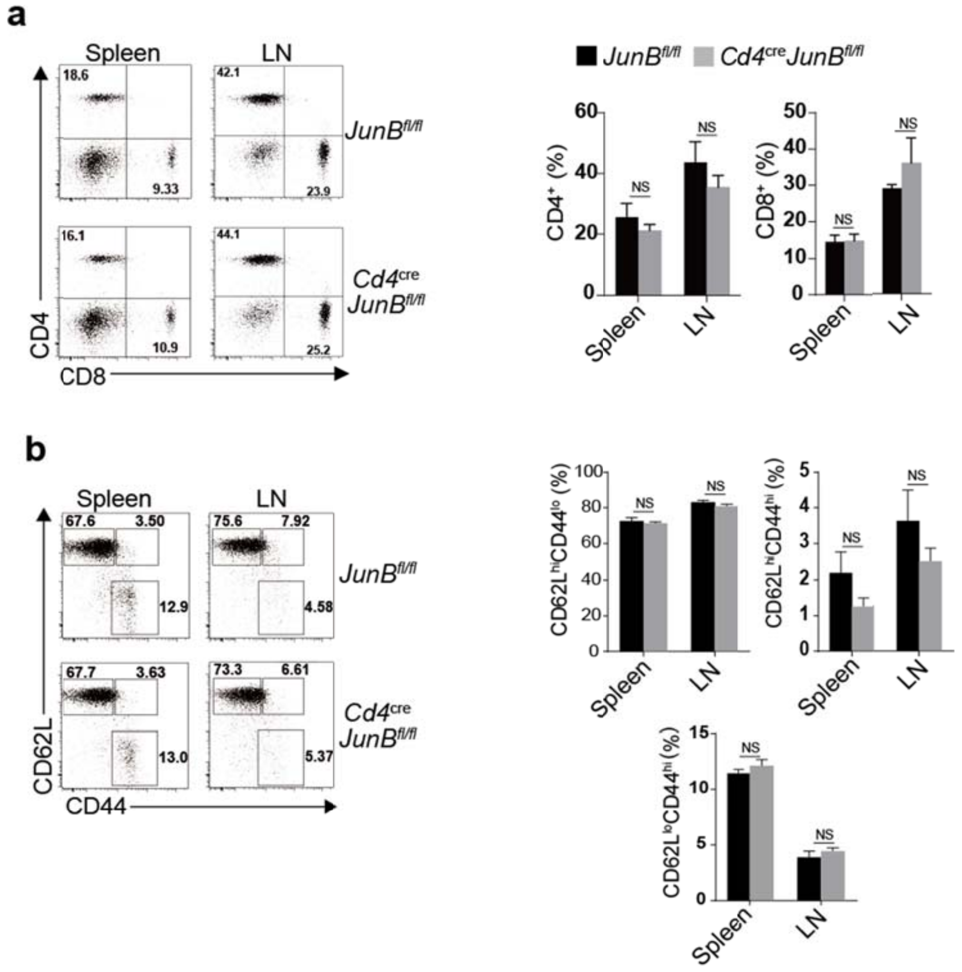

Supplementary Figure 3. Flow cytometry data showing cells expressing CD4 and CD8 in lymphocytes (**a**) or CD62L and CD44 in CD4$^+$ cells (**b**) in LNs and spleens of $Cd4^{Cre}JunB^{fl/fl}$ and control $JunB^{fl/fl}$ mice. NS, not significant differences (p>0.05) by unpaired two-tailed Student’s t test. Data represent two independent experiments.

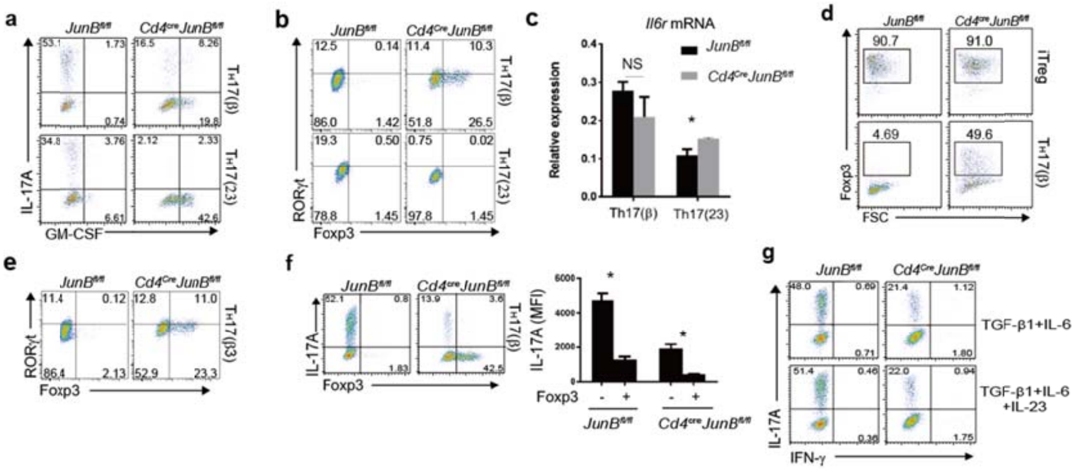

Supplementary Figure 4. (**a, b**) Naive CD4$^+$ T cells from $Cd4^{Cre}JunB^{fl/fl}$ and control $JunB^{fl/fl}$ mice were activated under $T_H17(\beta)$- or $T_H17(23)$-polarizing conditions for 3 days, and expression of IL-17A and GM-CSF (**a**) or RORγt and Foxp3 (**b**) was analyzed by flow cytometry. (**c**) Naive CD4 T cells were activated under $T_H17(\beta)$ or $T_H17(23)$ conditions for 3 days. *Il6r* mRNA expression was measured by qRT-PCR. Data were normalized to *Hprt* mRNA. Error bars indicate s.d. (n=3). Asterisks indicate significant differences ($P<0.05$) by unpaired two-tailed Student's t test. NS, not significant. (**d**) Naive CD4$^+$ T cells were activated under $T_H17(\beta)$- or iTreg-polarizing conditions for 3 days. Foxp3 expression was analyzed by flow cytometry. (**e**) Naive CD4$^+$ T cells were activated in the presence of TGF-β3 and IL-6 ($T_H17(\beta3)$) for 3 days, and expression of RORγt and Foxp3 was analyzed by flow cytometry. (**f**) Naive CD4$^+$ T cells were activated under $T_H17(\beta)$ conditions for 3 days, and expression of IL-17A and Foxp3 was analyzed by flow cytometry. The right graph shows mean of fluorescence intensity (MFI) of IL-17A expression. Error bars indicate s.d. (n=3). Asterisks indicate significant differences ($P<0.05$) by unpaired two-tailed Student's t test. (**g**) Naive CD4$^+$ T cells were activated in the presence of TGF-β1 and IL-6 with or without IL-23 for 3 days. Expression of IL-17A and IFN-γ was detected by flow cytometry. (**a-g**) Data represent two independent experiments. (**h**) Gating strategy for flow cytometry analysis in Fig. 2e. Dead cells were stained with Zombie dye.

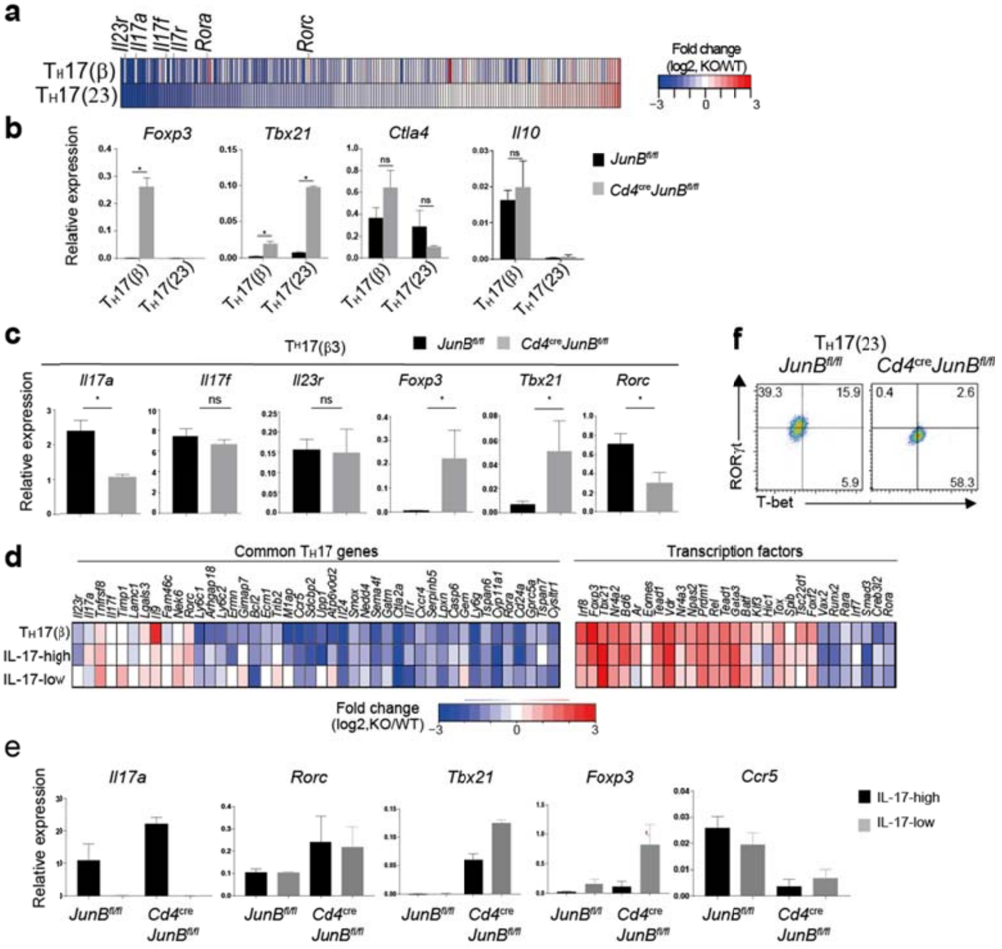

Supplementary Figure 5. (**a**) Effect of JunB deficiency on expression of all common T_H17 genes that are significantly upregulated in both T_H17(β) and T_H17(23) compared to T_H0. Heat map data show fold changes of expression in *Cd4*^{Cre}*JunB*$^{fl/fl}$ (KO) versus control (WT) cells as in Fig. 3c. (**b, c**) Naive $CD4^+$ T from *Cd4*^{Cre}*JunB*$^{fl/fl}$ and control mice were activated under T_H17(β), T_H17(23) (**b**) or T_H17(β3) (**c**) conditions for 84 hr, and mRNA expression of indicated genes was analyzed by qRT-PCR. (**d**) Naive $CD4^+$ T cells were activated under T_H17(β)-polarizing conditions for 60 hr, and IL-17-high and IL-17-low populations were sorted by FACS and used for microarray analysis. Heat map data show fold changes of expression in *Cd4*^{Cre}*JunB*$^{fl/fl}$ (KO) versus control (WT) cells for common T_H17 genes and genes categorized as transcription factors. Only genes that showed a significant change in *Cd4*^{Cre}*JunB*$^{fl/fl}$ cells compared to controls were shown. (**e**) qRT-PCR analysis of IL-17-high and IL-17-low populations in *JunB*-deficient or control cells activated under T_H17(β) conditions. (**f**) Flow cytometry analysis of cells activated under T_H17(23) conditions for 3 days. T-bet and RORγt expression was detected. (**b**, **c**, **e**) Error bars indicate s.d. (n=3). Asterisks indicate significant differences ($p<0.05$) by unpaired two-tailed Student's t test. ns, not significant. (**a-e**) Data represent two independent experiments.

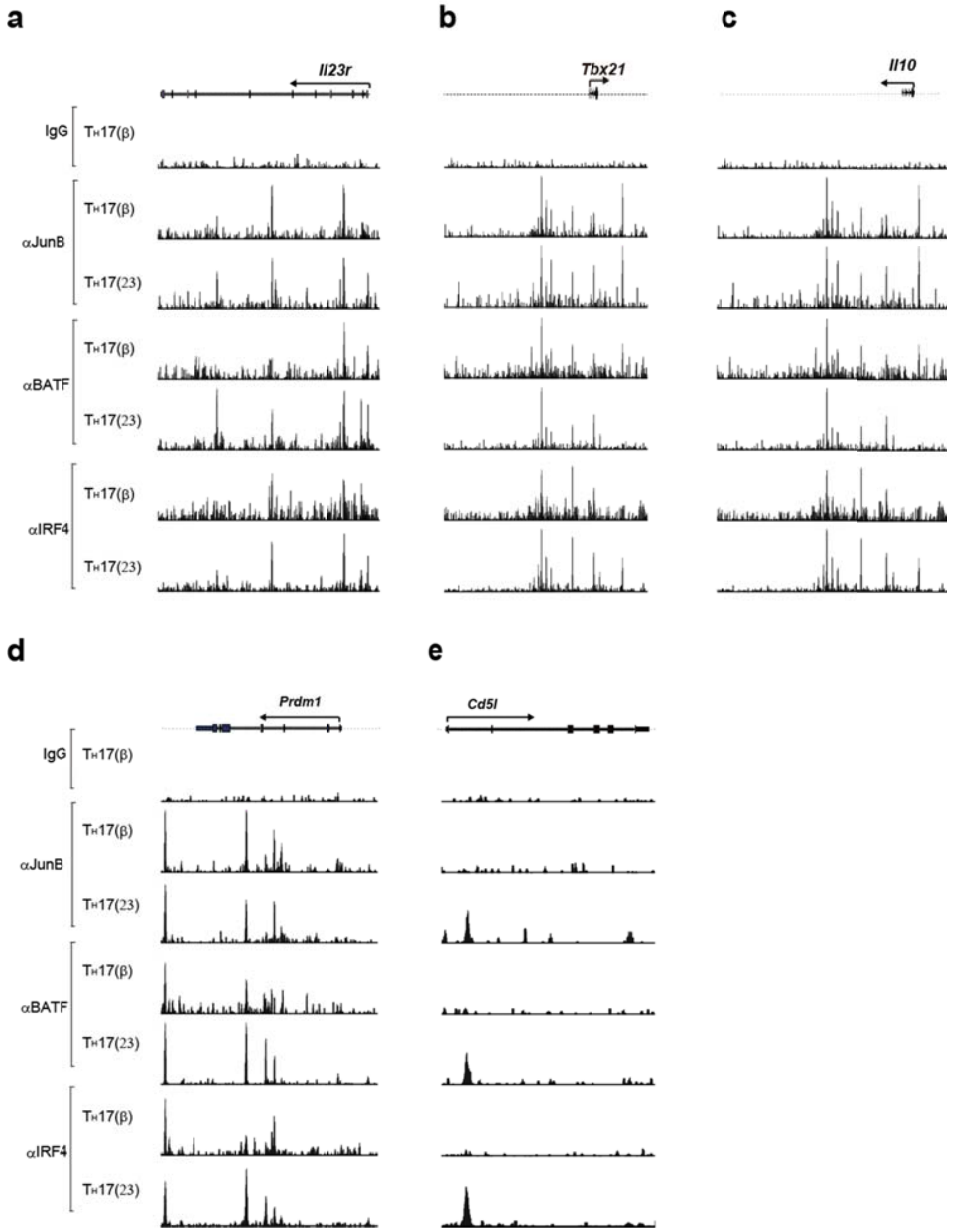

Supplementary Figure 6. Naive CD4$^+$ T cells from wild-type mice were activated under $T_H17(\beta)$- or $T_H17(23)$-polarizing conditions for 60 hr and subjected to ChIP-seq analysis using JunB, BATF, and IRF4 antibodies. *Il23r* (**a**), *Tbx21* (**b**), *Il10* (**c**), *Prdm1* (**d**), *Cd5l* (**e**) loci are shown. Schematics representations at the top of panels indicate exons (black boxes), and introns (solid lines). Data represent two independent experiments.

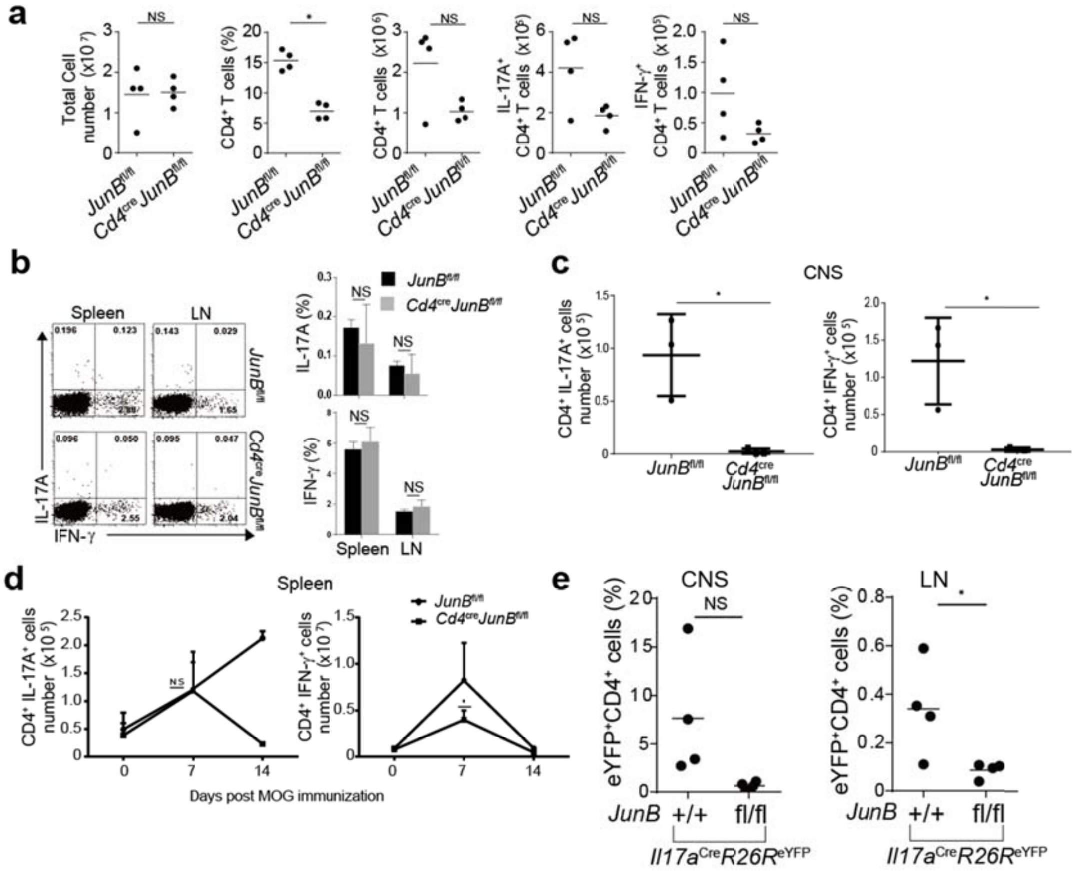

Supplementary Figure 7. (**a**) Absolute numbers of $CD3^+CD4^+$ cells expressing IL-17A, IFN-γ, RORγt, and Foxp3 were determined using flow cytometry data in Fig. 5. (**b**) Flow cytometry analysis of cells expressing IL-17A and IFN-γ in $CD4^+$ cells in LNs and spleens of *Cd4*^{Cre}*JunB*$^{fl/fl}$ and control *JunB*$^{fl/fl}$ mice. Data represent at least two independent experiments. (**c**) Absolute numbers of $CD3^+CD4^+$ cells expressing IL-17A and IFN-γ were determined using flow cytometry data in Fig. 6c. (**d**) Absolute numbers of $CD3^+CD4^+$ cells expressing IL-17A or IFN-γ in spleens of EAE-induced mice. (**e**) Frequencies of $CD4^+$ $EYFP^+$ cells in LNs and CNS of *Il17a*^{Cre}*R26R*^{eYFP}*JunB*$^{fl/fl}$ or control *Il17a*^{Cre}*R26R*^{EYF}*JunB*$^{+/+}$mice on day 14 after EAE induction (as in Fig. 6d). (**a-e**) Error bars indicate s.d. n=3 (**a**, **b**, **d**), n=4 (**c**, **e**). Asterisks indicate significant differences ($p<0.05$) by unpaired two-tailed Student's t test. NS, not significant. Data represent two independent experiments.

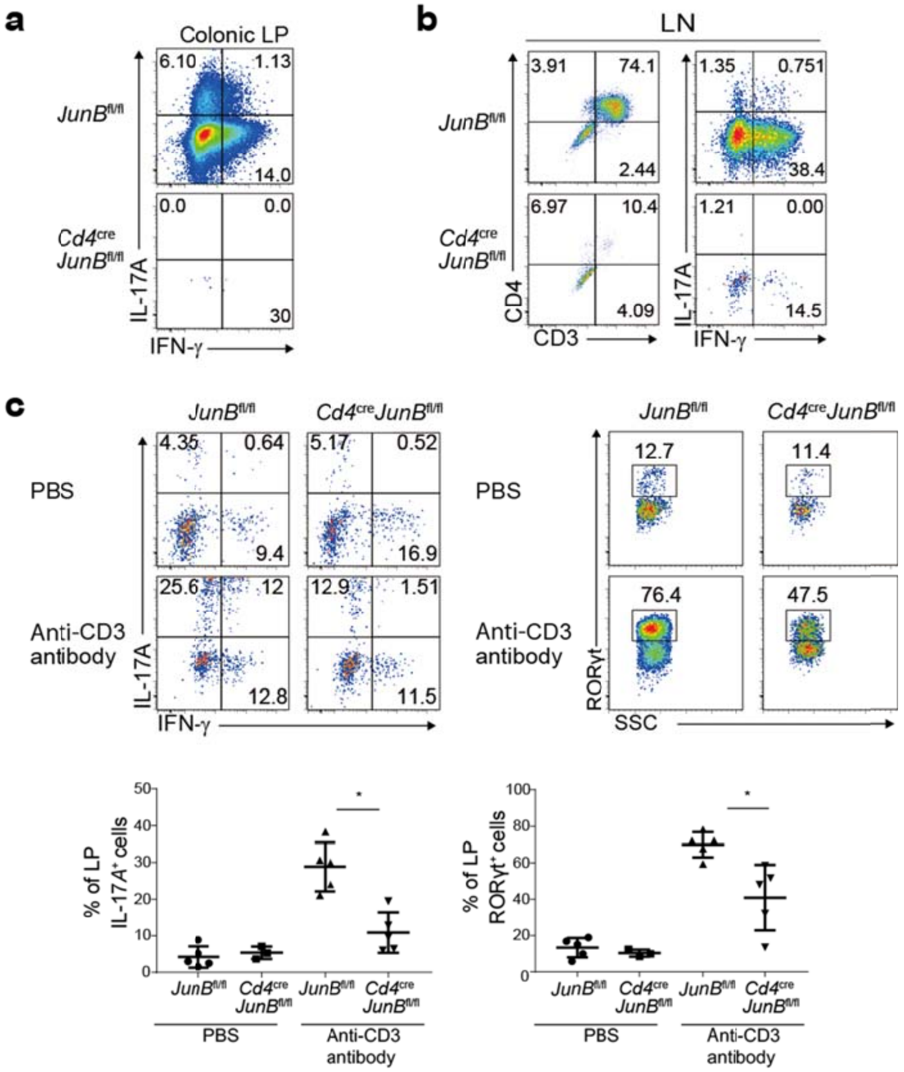

Supplementary Figure 8. (a, b) Colitis was induced in *Rag1*-deficient mice by transferring CD4^{+}CD45RB^{hi}CD25^{-} cells from *Cd4^{Cre}JunB$^{fl/fl}$* or control *JunB$^{fl/fl}$* mice. Frequencies of CD4^{+} T cells expressing IL-17A and IFN-γ in colonic LP (**a**) and LNs (**b**) were analyzed. Data represent at least two independent experiments. (**c**) Mice (n=5) were injected with anti-CD3 antibody three times at 0, 48, and 96 hr. At 4 hr after the final injection, cells were isolated from LP of duodenums and used for flow cytometry analysis (gated on CD4^{+} and TCRβ^{+}). Asterisks indicate significant differences ($p<0.05$) by unpaired two-tailed Student's t test. NS, not significant. Data represent two independent experiments.

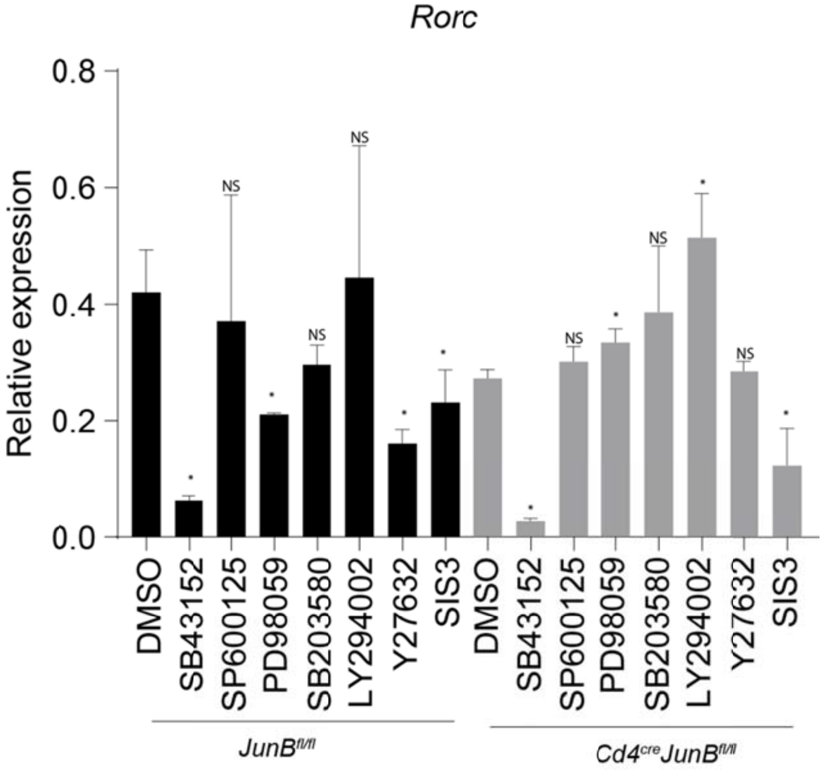

Supplementary Figure 9. Naive CD4+ T cells from *Cd4^{Cre}JunB$^{fl/fl}$* or control *JunB$^{fl/fl}$* mice were activated under $T_H17(\beta)$ conditions in the presence of TGF-β1 receptor kinase inhibitor (SB43152), JNK inhibitor (SP600125), MEK inhibitor (PD98059), p38 inhibitor (SB203580), PI3 kinase inhibitor (LY294002), or ROCK inhibitor (Y27632), SMAD3 inhibitor (SIS3) or DMSO alone for 60 hr. *Rorc* mRNA levels were measured by qRT-PCR. Results were normalized to *Hprt* mRNA expression. Error bars indicate s.d. (n=3). Asterisks indicate significant differences ($p<0.05$) compared to DMSO-treated control samples by unpaired two-tailed Student's t test. NS, not significant. Data represent two independent experiments.

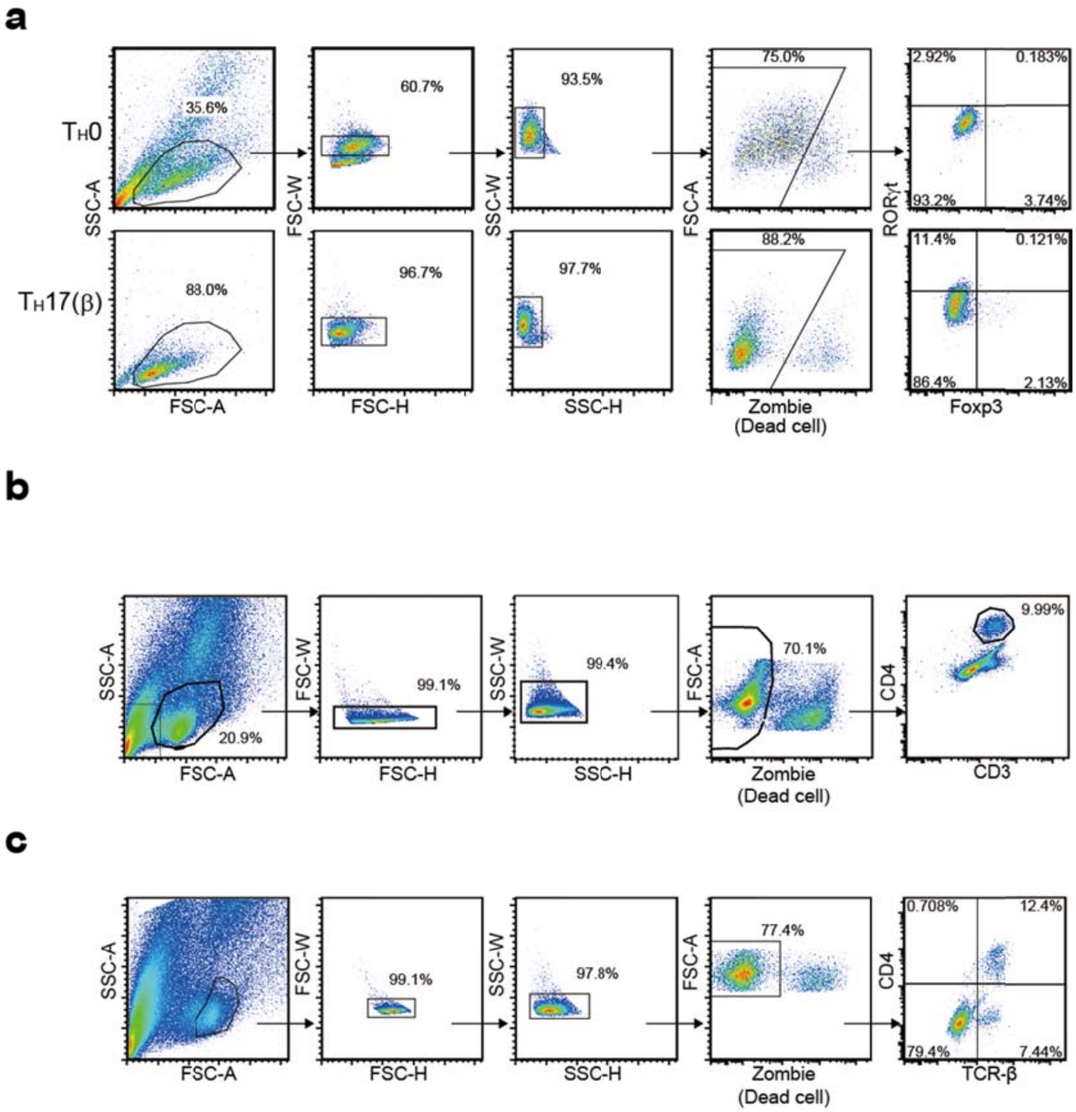

Supplementary Figure 10. Gating strategies for flow cytometry analyses of T cells differentiated *in vitro* related to Fig. 2 (**a**), T cells isolated from LNs, spleen and gut related to Figs. 5 and 6 (**b**), and duodenum T cells related to Supplementary Figure 8 (**c**).

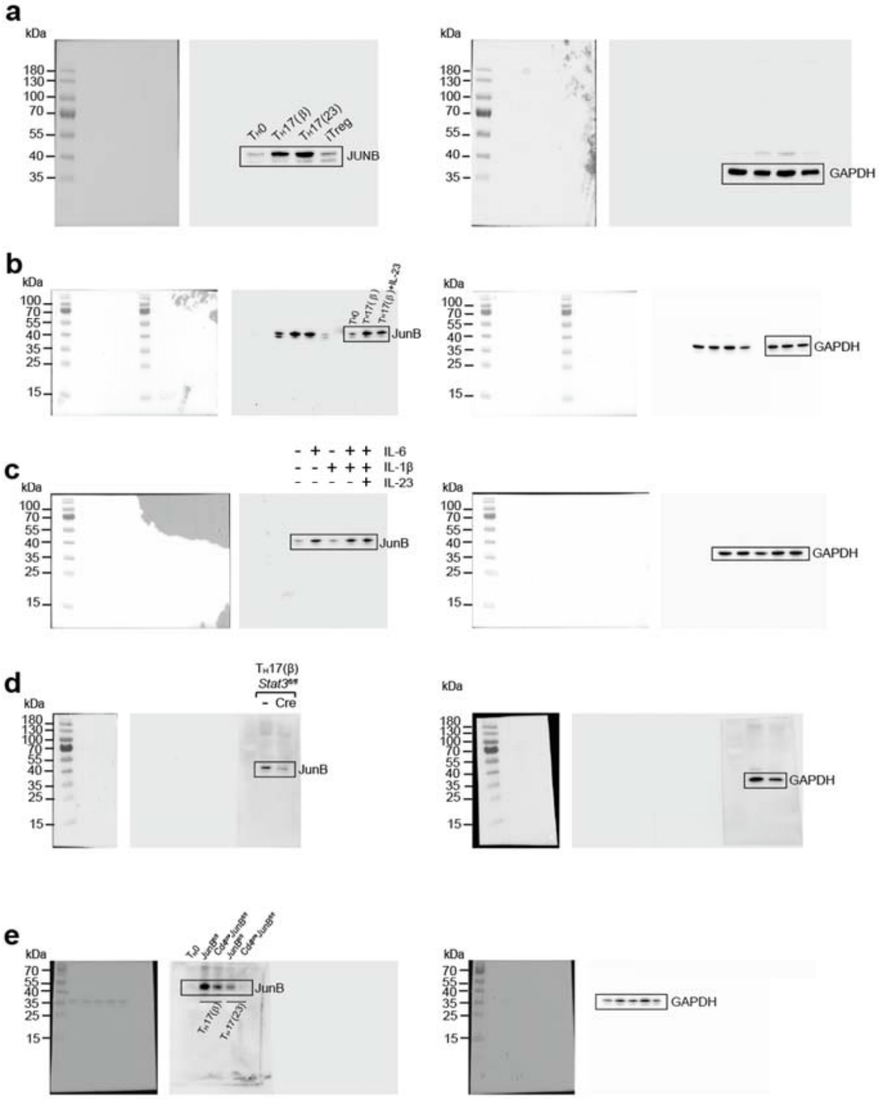

Supplementary Figure 11. Original data of immunoblots. (**a**) Immunoblot data corresponding to Fig. 1a. (**b**) Immunoblot data corresponding to Fig. 1c. (**c**) Immunoblot data corresponding to Fig. 1d. (**d**) Immunoblot data corresponding to Fig. 1e. (**e**) Immunoblot data corresponding to Fig. 2b.

Gene Name	Refelenced sequence	Primer	Sequence
Il17a qRT-PCR	NM_010552	F	CAGACTACCTCAACCGTTCCAC
		R	TCCAGCTTTCCCTCCGCATTGA
Il17f qRT-PCR	NM_145856	F	AACCAGGGCATTTCTGTCCCAC
		R	GGCATTGATGCAGCCTGAGTGT
Il23r qRT-PCR	NM_144548	F	GTCCACCAAACTTCCCAGACAG
		R	CCTGAAGCAGGATGTCCTCTGA
Enpp2 qRT-PCR	NM_001136077	F	GGATTACAGCCACCAAGCAAGG
		R	ATCAGGCTGCTCGGAGTAGAAG
Foxp3 qRT-PCR	NM_001199347	F	CCTGGTTGTGAGAAGGTCTTCG
		R	TGCTCCAGAGACTGCACCACTT
Tbx21 qRT-PCR	NM_019507	F	CCACCTGTTGTGGTCCAAGTTC
		R	CCACAAACATCCTGTAATGGCTTG
Rorc qRT-PCR	NM_001293734	F	GTGGAGTTTGCCAAGCGGCTTT
		R	CCTGCACATTCTGACTAGGACG
Hprt qRT-PCR	NM_013556	F	GAAGAGCTACTGTAATGATCAGTCAAC
		R	AGCAAGCTTGCAACCTTAACCA
JunB qRT-PCR	NM_008416	F	GACCTGCACAAGATGAACCACG
		R	ACTGCTGAGGTTGGTGTAGACG
Rorc region (+10,747-10,824) ChIP-PCR	NC_000069	F	GGGCCCTGAGATGGTAAGTT
		R	GGGTGCTGAGTAATCACAGGA
Il17a region (+9,823-9,921) ChIP-PCR	NC_000067	F	GGCTTGTCCCTCACATACCT
		R	ATATGGGCATGAGCAAAGTG
Ctla4 region (+4,498-4,625) ChIP-PCR	NC_000067	F	TGTTTGTGTGCTTCTGAGCAGGGT
		R	GGGTTGCCCAGAGACTGCTGTGT

Supplementary Table 1. Primers used in qRT-PCR.and ChIP-PCR
